# Summary version of the standards, options and recommendations for nonmetastatic breast cancer (updated January 2001)

**DOI:** 10.1038/sj.bjc.6601081

**Published:** 2003-08-15

**Authors:** L Mauriac, E Luporsi, B Cutuli, A Fourquet, J R Garbay, S Giard, F Spyratos, B Sigal-Zafrani, J M Dilhuydy, V Acharian, C Balu-Maestro, M P Blanc-Vincent, C Cohen-Solal, B De Lafontan, M H Dilhuydy, B Duquesne, R Gilles, A Lesur, N Shen, L Cany, I Dagousset, M H Gaspard, H Hoarau, A Hubert, M H Monira, N Perrié, G Romieu

**Affiliations:** 1 Institut Bergonié, Bordeaux, France; 2 Centre Alexis Vautrin, Nancy, France; 3 Polyclinique de Courlancy, Reims, France; 4 Institut Curie, Paris, France; 5 Institut Gustave Roussy, Villejuif, France; 6 Centre Oscar Lambret, Lille, France; 7 Centre René Huguenin, Saint-Cloud, France; 8 Institut Bergonié, Bordeaux, France; 9 Clinique les Cigognes, Pau, France; 10 Centre Antoine Lacassagne, Nice, France; 11 FNCLCC, Paris, France; 12 Institut Claudius Régaud, Toulouse, France; 13 Private Practica Lyon, France; 14 Polyclinique Nord, Bordeaux, France; 15 Polyclinique Francheville, Périgueux, France; 16 Private Practice Paris, France; 17 Clinique Claude Bernard, Albi, France; 18 CNRS Bordeaux, France; 19 CHU de Bordeaux, Bordeaux, France; 20 Centre Val d'Aurelle-Paul Lamarque, Montpellier, France

**Keywords:** non metastatic breast neoplasms, practice guideline

In France, there are 35 000 new cases of breast cancer each year. There are various areas where clinical research is active, including benign tumours and risk factors for malignant transformation, biological evolution of normal and cancerous cells, prevention, screening, diagnosis, therapeutic strategies, rehabilitation and maintenance of quality of life.

This document concerns the diagnosis and therapeutic management of nonmetastatic breast cancer, and does not cover screening or prevention. This is a summary of an update (Mauriac *et al*, 2001) of the version published in 1996 (Mauriac *et al*, 1996), in which sections concerning some particular situations and *in situ* carcinomas have not been included (these will be covered in a specific document). In addition, breast cancer with a genetic predisposition (BRCAX) will not be specifically covered in this document.

## METHODS

The ‘Standards, Options and Recommendations’ (SOR) project, which started in 1993, involves a collaboration between the Federation of French Cancer Centres (FNCLCC), the 20 French Regional Cancer Centres, several French public university and general hospitals, private clinics and cancer-learned societies. The main objective of the SOR project is to develop clinical practice guidelines which can be used to improve the quality of health care and outcomes for cancer patients. The methodology is based on a literature review, followed by critical appraisal by a multidisciplinary group of experts to produce draft guidelines which are then validated by specialists in cancer-care delivery. The details of the methodology used for developing these SOR have been described previously (Fervers *et al*, 2001).

A multidisciplinary working group was set up by the FNCLCC. References to pertinent articles were identified by the global bibliographic monitoring process of MEDLINE, set up in 1996 and by specific searches in other databases such as *Embase*®, *Cancerlit*® and the *Cochrane Library*®, and from the personal reference lists of the members of the working group. In addition, the French National Agency for Health Accreditation and Evaluation (ANAES) provided a complementary updated bibliographic search (1999 to October 2000). After selection and critical appraisal of these articles, the members of the working group drafted the SORs.

‘*Standards*’ identify clinical situations for which there exist strong indications or contraindications for a particular intervention and ‘*Options*’ identify situations for which there are several alternatives, none of which have shown clear superiority over the others (Table 1

**Table 1:** Definition of ‘Standards, Options and Recommendations’

Standards	Procedures or treatments which are considered to be of benefit, inappropriate or harmful by unanimous decision based on the best available evidence
	
Options	Procedures or treatments which are considered to be of benefit, inappropriate or harmful by a majority, based on the best available evidence
	
Recommendations	Additional information to enable the available options to be ranked using expect criteria (e.g. survival, toxicity) with an indication of the level of evidence

). In any SOR, there can be several ‘*Options*’ for a given clinical situation. ‘*Recommendations*’ enable the ‘*Options*’ to be weighed according to the available evidence. Several interventions can be recommended for the same clinical situation, so that clinicians can make a choice according to specific clinical parameters, for example, local circumstances, skills, equipment, resources and patient preferences. Adapting the SORs to a local situation is possible if the reason for the choice is sufficiently transparent and this is crucial for successful implementation. Inclusion of patients in clinical trials is an appropriate form of patient management in oncology and is recommended frequently within the SORs, particularly in situations where evidence is too weak to support an intervention.

The type of evidence underlying any ‘*Standard*’, ‘*Option*’ or ‘*Recommendation*’, is indicated using a classification developed by the FNCLCC based on previously published models. The level of evidence depends not only on the type and quality of the studies reviewed, but also on the concordance of the results (Table 2

**Table 2:** Definition of level of evidence

*Level A*
There exists a high standard meta-analysis or several high-standard randomised clinical trials which give consistent results

*Level B*
There exists good quality evidence from randomised trials (B1) or prospective or retrospective studies (B2). The results are consistent when considered together

*Level C*
The methodology of the available studies is weak or their results are not consistent when considered together

*Level D*
Either the scientific data does not exist or there is only a series of cases

*Expert agreement*
The data does not exist for the method concerned but the experts are unanimous in their judgement

). When no clear scientific evidence exists, judgement is made according to the professional experience and consensus of the expert group (‘expert agreement’).

The document containing the SORs was then reviewed by a group of independent experts (see the appendix) and after taking into consideration their comments, the guidelines were validated by the working group.

This document summarises the key recommendations from the complete updated edition that has been published in French as a monograph (Mauriac, *et al*, 2001) and a French summary version (Mauriac *et al*, 2002) and is also available at the Internet address: http://www.fnclcc.fr.

## CLINICAL DIAGNOSIS (FIGURE 1 AND FIGURE 2)

### Diagnostic work-up of an abnormality in the breast

#### Clinical examination

**Figure 1:**
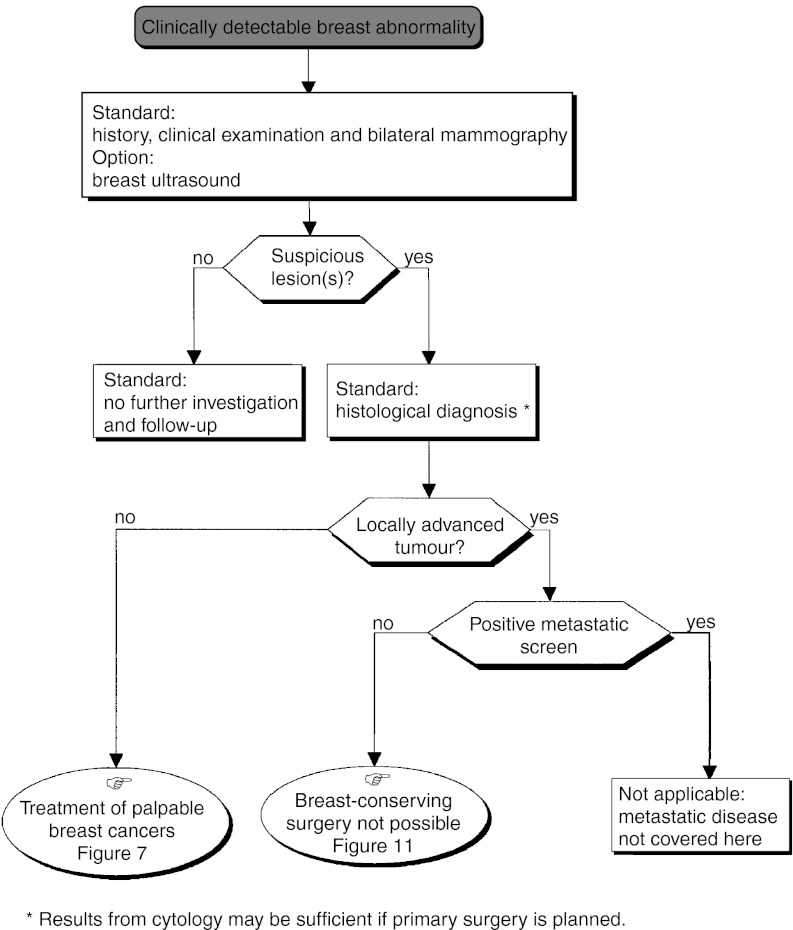
Diagnosis–clinically detectable breast abnormality.

**Figure 2:**
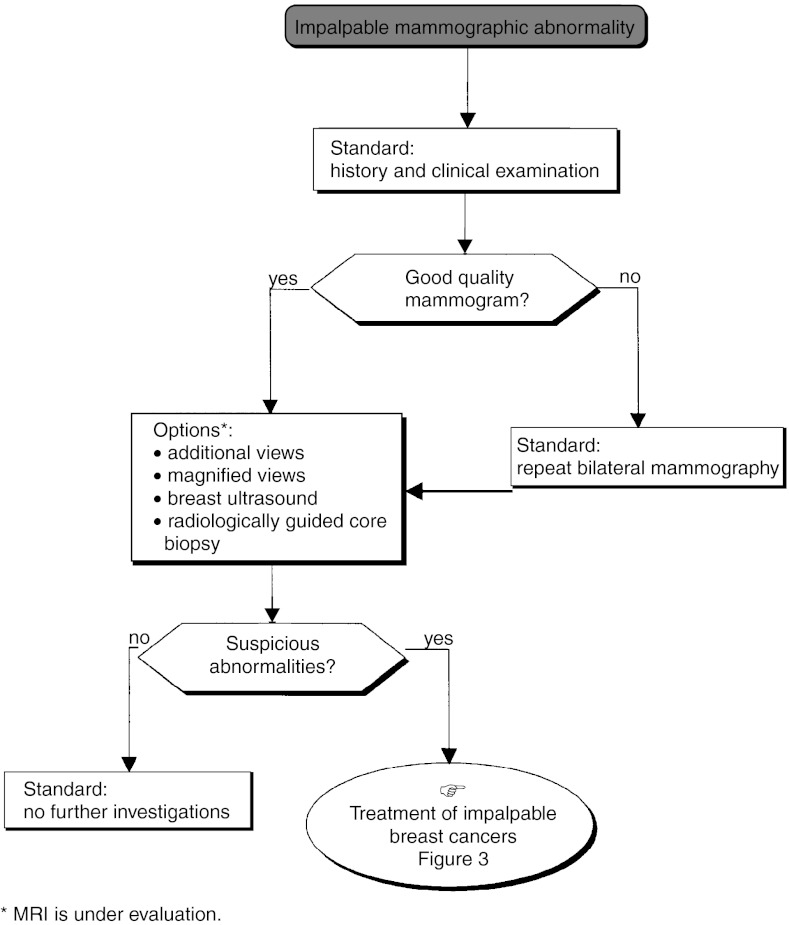
diagnosis–impalpable mammographic abnormality.

In patients with locally and regionally advanced disease, clinical examination is likely to be of greater diagnostic value than in patients with less advanced disease (standard). Tumours with the following characteristics are unlikely to be operable, and are associated with a poor short-term prognosis: inflammatory change, deep extension, lymphadenopathy, breast oedema and/or lymphoedema in the upper limbs. These factors also indicate an increased risk of locoregional and metastatic recurrence (standard). Thus, nonoperability is not always due to difficulties associated with the surgery itself. Surgery may still be appropriate, however, to achieve local control, despite a poor prognosis. Imaging can be used to confirm findings from clinical examination. In some cases, clinical examination will be normal, so that the diagnosis will only be possible following imaging (standard).

### Diagnostic imaging

A standard bilateral mammogram with two views (front and external oblique), can be complemented by other views and by ultrasonography (standard). The equipment used for mammography should undergo regular quality control (standard). To confirm the diagnosis, additional views and enlargements may be necessary (option).

Specific imaging is not indicated in the examination of lymphadenopathy. Standard reporting of mammograms should be used in which the stated diagnosis is based on the mammographic features suspicious of malignancy using the American College of Radiology classification (recommendation).

The diagnostic findings should be discussed by a multidisciplinary team. When the level of suspicion from imaging justifies histological verification, this should be done using interventional breast diagnostic techniques in an outpatient setting under a local anaesthetic.

### Pretreatment diagnosis

The diagnosis of malignancy can be obtained from cytology (fine-needle aspiration) or tissue sample (core biopsy), whereas the diagnosis of an invasive carcinoma can only be made from a biopsy sample (standard).

The histological diagnosis of impalpable lesions should be compared with the diagnostic hypotheses generated from diagnostic imaging (recommendation). The diagnosis of ductal carcinoma *in situ*, lobular carcinoma *in situ* and atypical hyperplasia from a needle biopsy sample should always be reconfirmed using a sample obtained by surgical excision (recommendation, level of evidence: B1). Regular assessment of the diagnostic performance of image-guided biopsy systems is recommended (recommendation, expert agreement).

## PATHOLOGICAL EXAMINATION AND CLASSIFICATION

### Frozen sections

Examination of frozen sections is not indicated for isolated clusters of microcalcification or for a tumour measuring less than 10 mm (standard). It is indicated in other cases where it is likely to modify the surgical plan (standard). This examination should not jeopardise the quality of the excised sample and therefore the reliability of the final histological diagnosis (standard).

### Surgical margins

The standard histological report should mention the findings from the diagnostic work-up and all factors necessary for establishing the prognosis:

- the size of the malignant lesion(s) (in mm);
- the histological type;
- the histological grade (specifying the grading system used);
- the percentage of any ductal carcinoma *in situ* (DCIS);
- the presence of peritumoral vascular invasion;
- the status of the surgical margins, including that of all additional excised samples.

For an invasive tumour, the measurement of the infiltrating component should be given, including data from macroscopic and microscopic examinations (recommendation). The overall size of the lesions (including the invasive component and any associated intraductal component) can be documented (option, expert agreement).

If there is intraductal carcinoma only, the size of the lesions should be assessed by combining the findings from the radiological and histopathological examinations (recommendation). If this assessment is difficult (multiple foci, etc.), the number of ‘positive’ sections out of the total number of sections examined should be stated (recommendation, expert agreement).

The quality of the excised sample is defined by two criteria (recommendation):

- the distance (in mm) between all malignant foci and the nearest excision margin (identified using surgical guide marks);
- the nature of the tumour lesion (invasive or intraductal) nearest to the surgical margin.

In cases where the surgical margin is invaded by tumour, the extent of this should be specified (recommendation, expert agreement).

### Mastectomy

The standard histological report for a mastectomy sample should state the details of the diagnostic work-up and those factors required to establish the prognosis:

- the sites and number of malignant lesions;
- the size of the malignant lesions (in mm);
- the histological type of the tumour;
- the histological grade (specifying the grading system used);
- the percentage of DCIS, if present;
- the presence of peritumoral vascular invasion;
- the presence of extension into the nipple (specifying position and type: intraductal, infiltrating, Paget's disease);
- the presence if any of cutaneous or pectoralis major fasciomuscular involvement.

For an invasive tumour, the extent of the invasive component should be given, integrating data from macroscopic and microscopic examinations (recommendation). The overall extent of the lesions (including the invasive component and any associated intraductal component) can be documented (option, expert agreement).

If there is intraductal carcinoma only, the size of the lesions should be assessed by integrating the data from radiological and microscopic examinations (recommendation). If this assessment is difficult (multiple foci, etc.), the number of ‘positive’ sections out of the total number of sections examined should be given (recommendation, expert agreement).

### Axillary dissection

All lymph nodes removed by axillary dissection should undergo full histological examination using a series of macroscopic sections (standard).

The standard histological report for the samples from axillary dissection should specify:

- the number of lymph nodes examined;
- the number of metastatic lymph nodes, including the presence of micrometastases and if a sentinel node biopsy was performed before axillary dissection (standard);
- the number of metastatic lymph nodes with capsular rupture.

Optimal quality for axillary examination requires sampling from at least 10 lymph nodes (standard, level of evidence: B1). This is usually achieved by a level I/II axillary dissection (standard, level of evidence: B1). Surgical guide marks should be made to orientate the sample, at least at one end (standard). The technique of sentinel node biopsy may avoid axillary dissection in 85–90% of patients without lymph node involvement (level of evidence: B1), but cannot be recommended until the results of ongoing studies are available (recommendation, expert agreement). This technique requires input from a multidisciplinary team experienced in the area (recommendation, level of evidence: B1).

### Histological classification of breast cancer

The standard histological classification of breast cancer is that developed by the World Health Organisation (WHO) (standard). The standard for histo-prognostic grading is that developed by Elston and Ellis (standard). This is applicable to all invasive cancers with the exception of medullary carcinomas and *in situ* carcinomas (standard). The coding of lesions can be performed using the CIMO/SNOMED system (WHO classification) and/or the ADICAP system (French classification) (option).

## INVESTIGATIONS FOR THE DETECTION OF METASTATIC DISEASE

There is no indication to undertake a metastatic screening before the confirmation of a diagnosis of an invasive carcinoma (standard). This is also true for *in situ* carcinomas (standard).

If a mastectomy is planned, a metastatic screening should be performed prior to surgery to avoid unnecessary surgery in women who already have metastatic disease, even if the probability is low (recommendation). In the absence of symptoms, a metastatic screening should only be performed after evaluation of the metastatic risk factors (see section on Prognostic factors) (recommendation). Assays of CA 15.3 and other tumour markers should not be done during the initial work-up because of their low sensitivity (standard, level of evidence: B2). At a more advanced stage of disease, they are often elevated but have no diagnostic value. Tumour markers can sometimes be used as a reference when poor prognostic factors are present (option). No study has shown an independent prognostic value for tumour markers. Nonspecific markers, such as CA 125, CA19-9 and TPA, should not be assayed (standard).

## PROGNOSTIC FACTORS

### Predictive factors for lymph node involvement

It is essential to perform a clinical examination of the axillary region even though it is imprecise (standard, level of evidence: B1). Lymph node involvement generally progresses towards the apex of the axilla (standard, level of evidence: B1). Tumour size is the principal risk factor for lymph node involvement (standard, level of evidence: B1). Histologically confirmed axillary involvement, tumour size and young age are the main predictive factors for internal mammary node involvement (standard, level of evidence: B2). The influence of the tumour site is controversial.

### Predictive factors for local breast recurrence

The most important clinical factors are young age (under 35 or 40 years old) and premenopausal status. The most important histological factors are: positive excision margins and the presence of an extensive ductal *in situ* component associated with the invasive component, high-grade tumour, the presence of peritumoral vascular invasion and inappropriate treatment.

### Predictive factors for metastatic disease

The most important clinical factors are young age (under 35 or 40 years old), tumour size and axillary node involvement. The histological factors with the strongest prognostic value are tumour size, histologically confirmed node involvement, the number of axillary nodes involved (⩾4), a high-grade tumour and positive excision margins. The presence of peritumoral vascular invasion is also a predictive factor for metastatic disease.

### Predictive factors for therapeutic response

Hormone receptor status should be determined in all cases of invasive disease (standard), using either a biochemical or immunohistochemical method (standard). The analysis for overexpression of c-erbB-2 should not be performed on a routine basis (standard). The predictive factors for response to chemotherapy, including c-erbB-2, are in the process of being evaluated. Cellular proliferation should be assessed in all infiltrating tumours, using either the mitotic index, S phase fraction measurement or Ki67 immunohistochemical assay (recommendation, expert agreement). Quality control is mandatory in all laboratories irrespective of the prognostic test performed (standard).

## TREATMENT MODALITIES

### Surgery

Irrespective of the surgical technique used, tumour excision should be complete with negative margins and should be adapted to the tumour size and the breast volume (standard). The optimal size of excision to ensure negative margins has not been defined (standard).

When breast-conserving surgery is to be undertaken, the aesthetic result should be acceptable to the patient (standard). Central tumours can be treated in a conservative manner if excision is complete (sometimes requiring excision of the nipple–areolar complex) (option).

Modified radical mastectomy is equivalent to radical mastectomy in terms of local control and survival (standard). Breast-conserving treatments with lumpectomy followed by radiotherapy are equivalent to mastectomy in terms of local recurrence and survival (standard, level of evidence: A). Subcutaneous mastectomy should not be considered for invasive or noninvasive breast cancer (recommendation, expert agreement).

### Complications after axillary node dissection

There appears to be no technique available to assess the risk of sequelae (particularly lymphoedema) following surgery to this anatomically complex and strategic area where several different lymphatic regions converge (back, legs, anterior thoracic wall and breast). Early physiotherapy for the shoulder and the thoracic wall is by far the most significant preventative treatment. Informing patients of the best means of prevention remains the best means of preventing lymphoedema. Lymphatic drainage is not indicated as a preventative measure (recommendation).

### Radiotherapy

Radiotherapy reduces the risk of mortality due to breast cancer (standard, level of evidence: A). However, it may increase the risk of long-term cardiovascular mortality if an inappropriate technique is used. Breast, chest wall or lymph node irradiation should be performed with caution, with the aim of limiting the irradiation of healthy tissue, while satisfying quality assurance criteria (standard). The dose prescription is standardised (the International Commission on Radiation Units (ICRU)) (standard, level of evidence: A).

After breast-conserving surgery, breast radiotherapy should always be performed, using a minimum dose of 50 Gy in 25 fractions (standard, level of evidence: A). Breast irradiation after breast-conserving surgery significantly reduces the risk of local recurrence irrespective of the initial disease stage (standard, level of evidence: A). In women under 50 years old, a boost should be administered routinely to the tumour bed even when the margins are clear (standard, level of evidence: B). Guidelines from an expert committee of the French Society of Oncological Radiotherapy (SFRO, 1991) cover the choice of target volume for irradiation following breast-conserving surgery. After mastectomy, the benefit from chest wall radiotherapy is greater in patients with the highest number of risk factors (standard, level of evidence: A).

Irradiation of the internal mammary lymph nodes is indicated in all cases of axillary lymph node involvement (standard, level of evidence: B1) and when the tumour is medial or central (standard, expert agreement). Irradiation of the infra- and supraclavicular lymph nodes is indicated in the presence of axillary lymph node involvement (standard, level of evidence: B1). The omission of lymph node irradiation, as defined above, is only justified in the setting of a randomised clinical trial (standard, expert agreement).

The choice of immediate breast reconstruction should not jeopardise the optimal use of locoregional radiotherapy and systemic treatment (recommendation). After axillary dissection, radiotherapy to the axilla should be avoided as much as possible because of the increased risk of locoregional complications (standard, level of evidence: C).

### Chemotherapy

Anthracycline-containing polychemotherapy is currently the most commonly used regimen in France and is more efficacious than the CMF regimen: cyclophosphamide, methotrexate and fluorouracil (5-FU) (standard, level of evidence: A). This is supported by the conclusions from the NIH consensus conference (NIH, 2000).

Adjuvant chemotherapy improves progression-free survival and overall survival in patients with node-positive breast cancer and in certain patients without node involvement. Premenopausal women seem to benefit more than menopausal women.

Doxorubicin, epirubicin, 5-FU, cyclophosphamide and methotrexate used in combination every 3–4 weeks, with a maximum of six cycles, is the reference treatment. The optimal number of cycles (four to six cycles) is unknown. Chemotherapy should be started promptly. The efficacy of perioperative chemotherapy has not been clearly proved, and should only be undertaken in the setting of a randomised clinical trial. High-dose chemotherapy, with or without stem cell infusion, is under evaluation and cannot be considered as a therapeutic standard. The optimal dose for epirubicin remains to be determined. Taxanes have not yet been shown to offer any benefit as adjuvant or neoadjuvant treatment.

Induction or neoadjuvant chemotherapy is an option in operable breast cancer where first-line breast-conserving surgery is not possible, in the absence of multifocal lesions, and where the patient would prefer breast conservation (option). Compared with adjuvant therapy, induction or neoadjuvant therapy has no effect on survival but has been shown to avoid mastectomy in more than 50% of women. The risk of local recurrence is higher than with a primary mastectomy and the possibility of breast-conserving surgery is reduced. After neoadjuvant chemotherapy, locoregional treatment should be performed in the same manner as that used for first-line locoregional treatment (standard).

In premenopausal women, the combination of hormone therapy with adjuvant chemotherapy does not lead to a significant improvement in global survival or progression-free survival. This may be due to the low number of young women treated with tamoxifen and/or the lack of stratification for hormone receptor status in previous studies (standard, level of evidence: A). However, the practice was recommended in November 2000 by the NIH consensus conference (NIH, 2000).

### Hormone therapy

Treatment with adjuvant tamoxifen is beneficial, despite its side effects, irrespective of the patients’ age, if the tumour expresses oestrogen receptors (standard, level of evidence: A). Tamoxifen should not be prescribed to women with tumours that do not express oestrogen receptors (standard, level of evidence: A). The optimal duration for adjuvant hormone therapy with tamoxifen is 5 years at a dose of 20 mg day^−1^ (standard, level of evidence: A). Patients treated with tamoxifen should have regular gynaecological clinical examinations (recommendation, expert agreement). Additional examinations are not necessary in the absence of symptoms.

Neoadjuvant treatment with antioestrogens can be used in elderly women with slowly evolving hormone-sensitive tumours (option, level of evidence: B1). This should be followed when possible by optimal locoregional treatment (option, expert agreement). At present, antioestrogens cannot be considered as standard neoadjuvant treatment for initially operable tumours.

Hormone therapy can be administered for different reasons, depending on the patients’ age:

- suppression of ovarian function in women with ovarian activity and/or as an antioestrogen,
- antioestrogenic therapy in postmenopausal women.

In postmenopausal women, the combination of chemotherapy with an antioestrogen significantly improves progression-free survival and overall survival (standard, level of evidence: A). The ratio of efficacy (overall or recurrence-free survival) and risk (toxicity) should be considered when taking the decision to prescribe this combination. The efficacy/risk ratio favours treatment in women with major metastatic risk factors (recommendation, expert agreement).

Other hormone therapy (progestogens, aromatase inhibitors) should not be considered as adjuvant treatments except in the setting of a randomised clinical trial (standard).

### Breast reconstruction and additional treatments

When mastectomy is necessary to obtain local control and the patient would prefer immediate breast reconstruction, multidisciplinary consultation is essential to assess the need for locoregional (irradiation) or systemic (chemotherapy or hormone therapy) treatment. Breast reconstruction is not a cancer treatment but is an integral component of breast cancer care (standard). It can be performed immediately or be delayed, but should never interfere with the administration of other treatments (chemotherapy and/or radiotherapy) (standard). Poor prognosis is not a contraindication for breast reconstruction (standard). However, the patient's performance status and/or a high risk of recurrence of the disease can be relative contraindications (standard). The patient should participate in the final decision (standard). There are three main techniques for reconstruction: submuscular inplant, latissimus dorsi flap or TRAM (*transverse rectus abdominis myocutaneous*) pedicle flaps and microanastomosed free flaps (option).

If the patient requires radiotherapy but wants immediate breast reconstruction, autologous tissue techniques should be used. A prosthesis can be irradiated but the patient should be informed about the potential risks, particularly that of substantial contraction as seen in about 50% of patients.

## TREATMENT STRATEGY

The patient should participate in her treatment decisions at every stage.

### Treatment evaluation

Treatment evaluation involves both functional and aesthetic evaluation, consideration of possible side effects and the patients’ quality of life (standard). Painful scarring (leading to limited mobility of the arm/shoulder) and lymphoedema are the most frequently observed complications after surgery. The aesthetic result depends on the quality of the surgery and radiotherapy technique (standard). Aesthetic problems do not occur following chemotherapy alone, but are observed when chemotherapy is used concomitantly with radiotherapy (standard). Visual analogue scales of well being are useful for assessing quality of life. Fatigue has a significant impact on quality of life and can be related to treatment and/or a depressive reaction. This should always be taken into consideration by the physician and its importance should not be underrated. Questionnaires exist for assessing fatigue and quality of life (standard).

### Locoregional treatment

Surgery and/or radiotherapy are used for the locoregional control of the disease.

#### Management of impalpable tumours

The therapeutic strategy for ductal carcinomas *in situ* is not covered in this document. The therapeutic management of impalpable tumours is often a stepwise process guided by the histological results of the previous intervention. The patient should be informed right from the start about the risks associated with repeated interventions.

**Figure 3:**
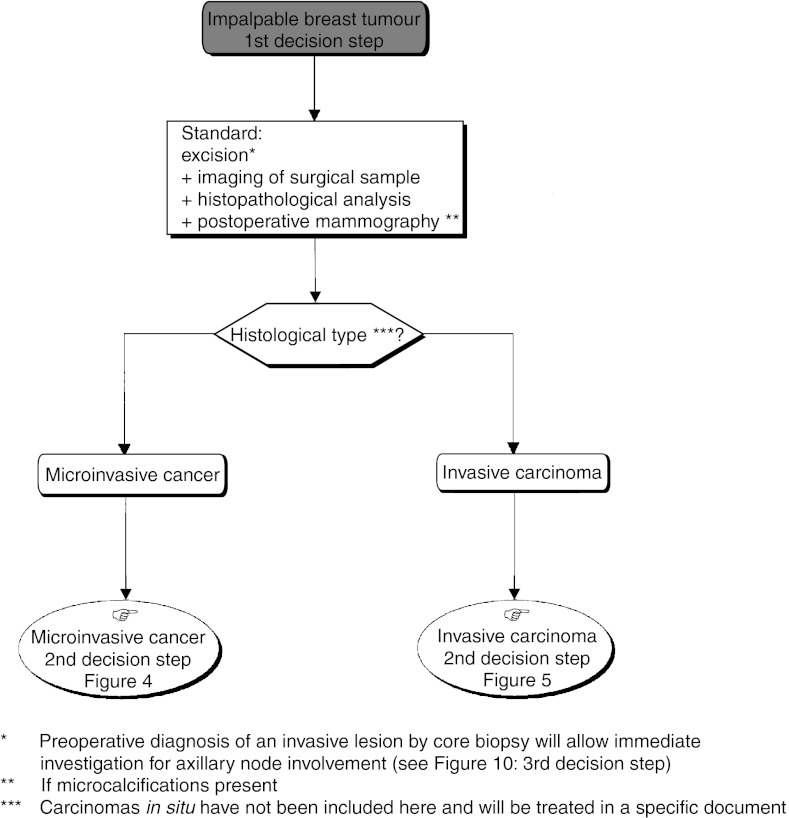
Impalpable operable tumour–locoregional treatment.

**Figure 4:**
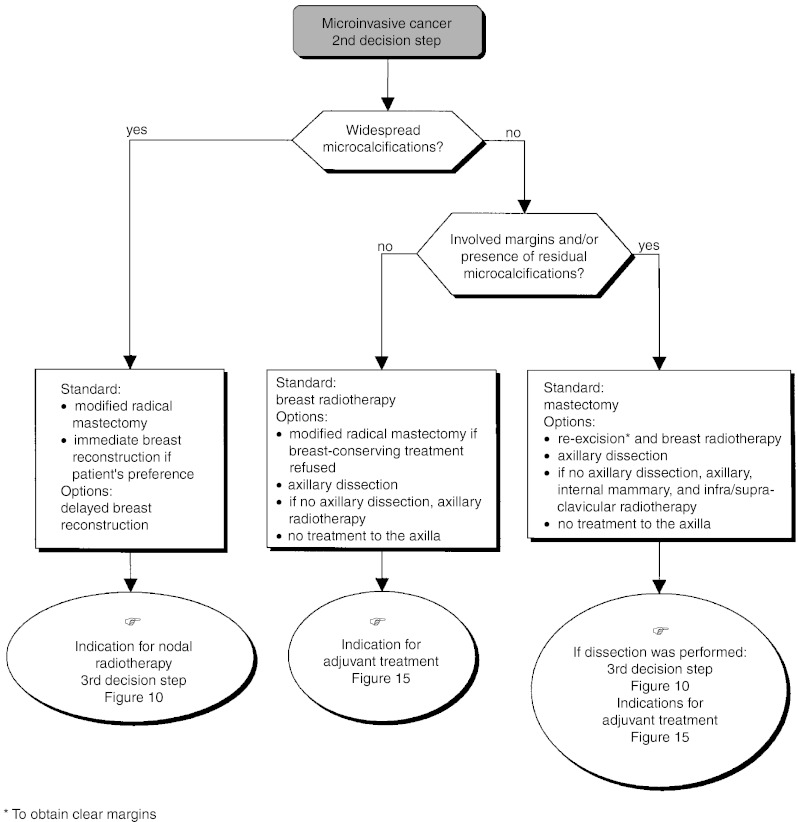
Operable impalpable tumour–locoregional treatment.

**Figure 5:**
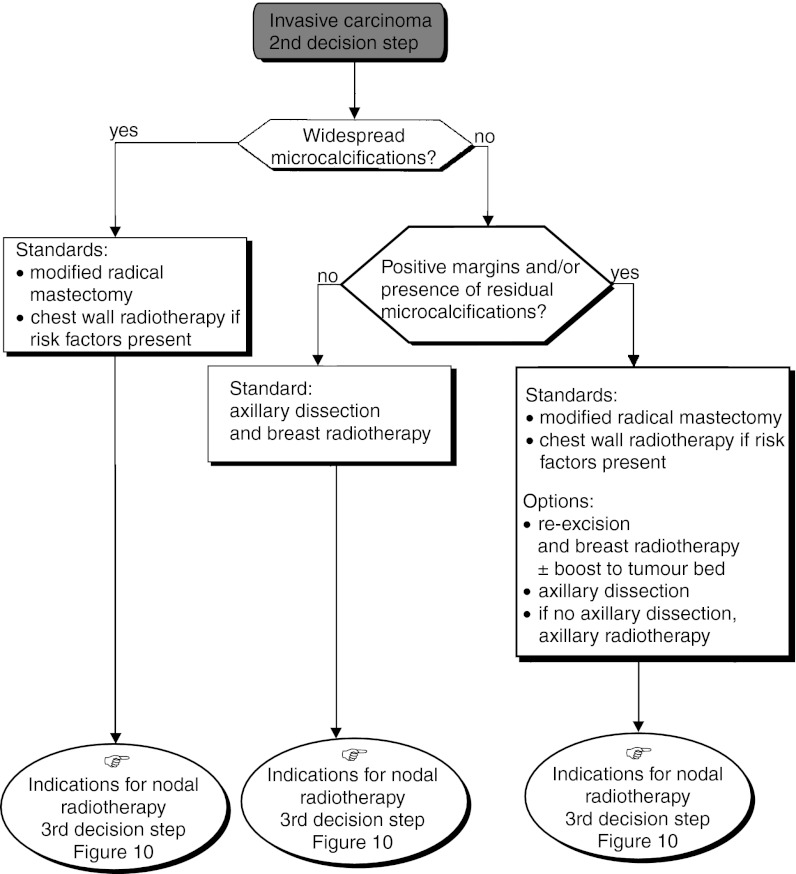
Operable impalpable tumour–locoregional treatment.

- *First decisional step (*Figure 3): This first step is diagnostic and potentially therapeutic and consists of a radiologically proven complete excision and histological analysis of the lesion (standard). In the absence of a palpable macroscopic lesion, a frozen section and primary axillary dissection should not be performed (recommendation, expert agreement). If microcalcifications are present, a mammogram should be performed 2 months after surgery (recommendation, expert agreement).
- *Second decisional step*: This step depends on the status of the surgical margins and the extent of the microcalcification before and/or after surgery.
  ○ *Microinvasive cancer (infiltrating component* ⩽*2 mm)*: There is no specific data concerning the risk of progression of microinvasive cancers. The recommendations are the same as those proposed for invasive cancers (Figure 4).
    – *Extensive microcalcifications at diagnosis* (*breast-conserving surgery inappropriate*): Modified radical mastectomy followed by immediate breast reconstruction should be offered (standard).
    – *Clear margins and no residual microcalcifications*: The breast should be irradiated (standard). Modified radical mastectomy can be considered if the patient refuses conservative treatment (option). Axillary dissection or axillary radiotherapy may also be performed (option). In the absence of known risk factors for metastatic recurrence, adjuvant systemic treatment should not be given (recommendation, level of evidence: B).
    – *Involved margins and/or residual microcalcifications*: Mastectomy is the standard treatment (standard). Re-excision and breast radiotherapy, or axillary dissection and radiotherapy can be performed (option). If re-excision does not provide clear margins, mastectomy should be performed (standard).
  ○ *Invasive cancer* (Figure 5):
    – *Extensive micro-calcifications at diagnosis*: Modified radical mastectomy is indicated (standard). In the event of lymph node invasion, radiotherapy to the chest wall and lymph nodes (internal mammary chain, infra- and supraclavicular) should be performed (standard).
    – *Clear margins and/or absence of residual microcalcifications*: The standard procedure is axillary dissection and breast radiotherapy (standard). If the patient presents with lymph node involvement, this should be followed by lymph node irradiation (internal mammary chain, infra- and supraclavicular) (standard). It is also possible to irradiate the lymph nodes without axillary surgery (option).
    – *Invaded margins and/or presence of residual microcalcifications*: Modified radical mastectomy should be performed (standard). In the event of lymph node involvement, this should be followed by the chest wall and lymph nodes (internal mammary chain, infra- and supraclavicular) irradiation (standard). Re-excision combined with axillary dissection, followed by breast radiotherapy can be undertaken. Breast radiotherapy with a boost to the tumour bed can also be considered (option).

#### Management of a single, palpable, Localised tumour treatable by breast-conserving surgery

**Figure 6:**
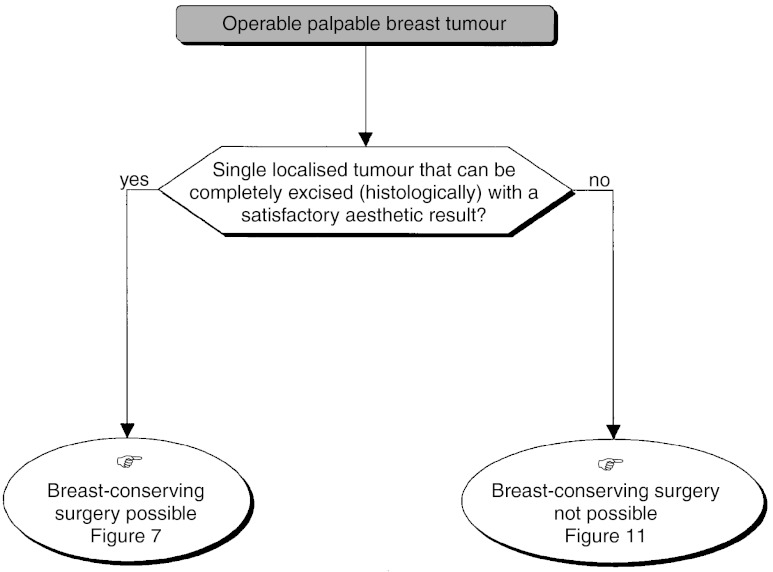
Operable palpable tumour–locoregional treatment.

A single palpable localised tumour can be completely excised with a wide margin (Figure 6

). This is dependent on the probability of achieving an excision with clear margins and a satisfactory aesthetic result.

**Figure 7:**
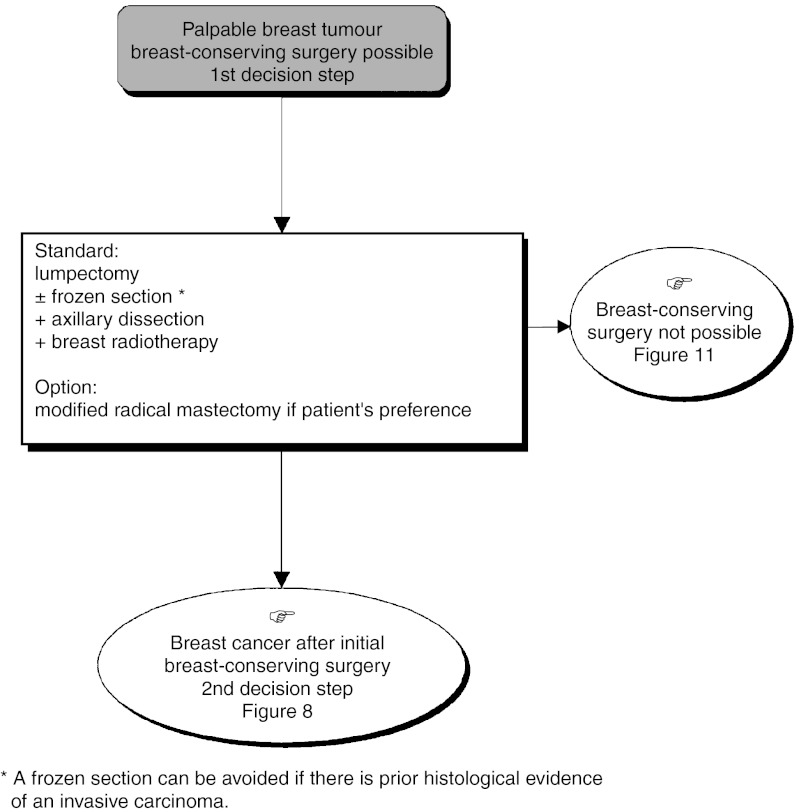
Operable palpable tumour–locoregional treatment.

**Figure 8:**
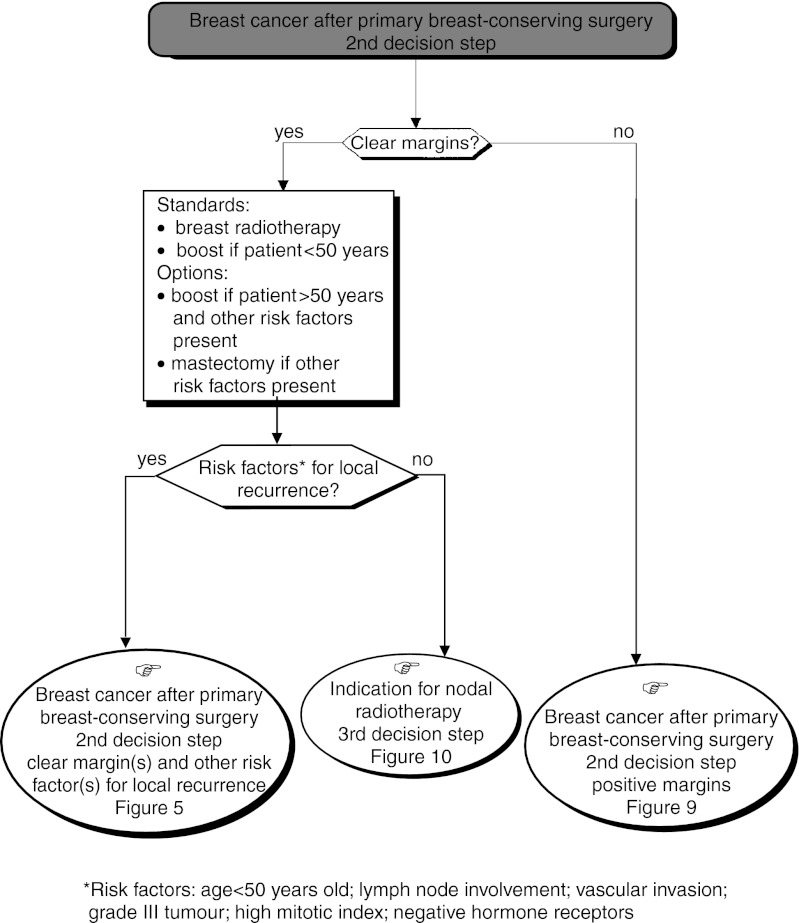
Operable palpable tumour–locoregional treatment.

**Figure 9:**
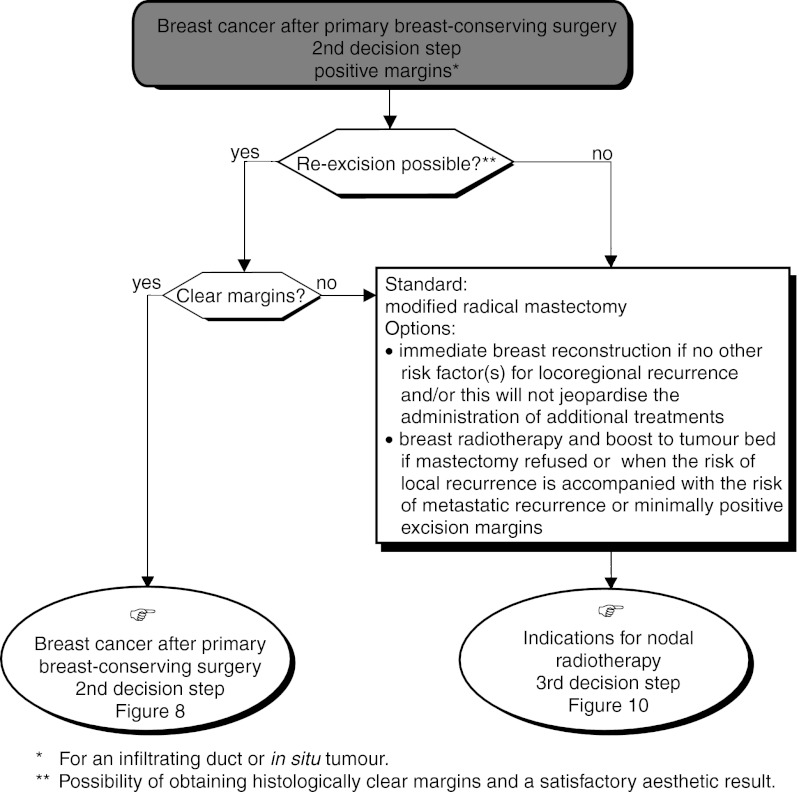
Operable palpable tumour–locoregional treatment.

- *First decision step* (Figure 7

): Lumpectomy (±frozen section), axillary dissection and breast radiotherapy is the standard. The breast should always be irradiated; this has been shown to reduce considerably the rate of local recurrence (recommendation, level of evidence. A). Axillary dissection should only be undertaken after the diagnosis of an invasive carcinoma has been confirmed (recommendation, expert agreement). It is essential to examine all tissue margins (recommendation). Central tumours can be managed with conservative treatment (recommendation, expert agreement). If the patient refuses conservative treatment, a modified radical mastectomy can be considered (option). If microcalcifications are present, a postoperative mammogram is essential to verify the presence or absence of residual lesions after conservative treatment (standard, expert agreement).
- *Second decision step* (Figure 8

): This step follows conservative treatment and depends on the histological status of the tissue margins and the presence of other risk factors.
  ○ *Clear excision margins* (Figure 8): Whole breast radiotherapy should be performed (standard) with an additional dose to the tumour bed (boost) if the patient is under 50 years old (standard). This association can be given to patients over 50 years old who have other risk factors for recurrence (option).
  ○ Positive excision margins (Figure 9

)
    – *When re-excision is possible (histologically clear margins possible and a satisfactory aesthetic result)*. The standard procedure is to undertake re-excision and breast radiotherapy (standard). This should be followed by a boost to the tumour bed if the patient is under 50 years old (standard) or has other risk factors (option). If a boost to the tumour bed and/or re-excision is performed, the aesthetic result should be satisfactory (recommendation, expert agreement). If the patient refuses re-excision, breast radiotherapy with a boost to the tumour bed can be considered (option). Modified radical mastectomy can also be considered (option).
    – *When re-excision is impossible (the margins will be involved after re-excision, and/or the aesthetic result will not be satisfactory)*. Modified radical mastectomy should be performed (standard). Immediate breast reconstruction can be considered if there are no other risk factors for locoregional recurrence and/or this does not prejudice the administration of additional treatment (option)..

If the patient refuses mastectomy and there is a high risk of metastatic recurrence or if there is minimal invasion of the excised tissue margins, breast radiotherapy and a boost to the tumour bed can be proposed (option, level of evidence: D).

**Figure 10:**
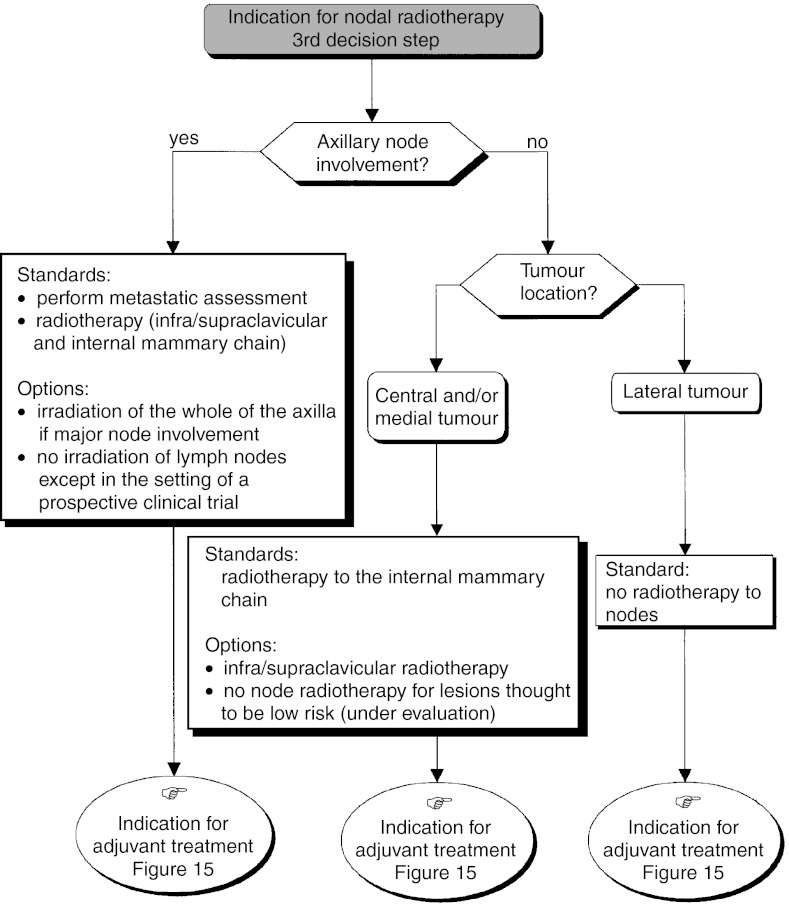
Operable tumour–indication for nodal radiotherapy.

- *Third decision step* (Figure 10

): This depends on the extent of axillary node involvement.
  ○ *Absence of axillary node involvement*: The decision depends on the tumour localisation.
    – *Lateral tumour* The nodal areas should not be irradiated (standard).
    – *Central and/or medial tumour* The internal mammary chain should be irradiated (standard). Infra- and supraclavicular radiotherapy can also be considered (option). Nodal irradiation for low-risk tumours is not recommended, and is currently under evaluation. – Irrespective of the tumour localisation, axillary radiotherapy should not be performed in the absence of histologically proven nodal involvement (recommendation, level of evidence: A).
  ○ *Presence of axillary node involvement*: Screen for distant metastases (standard) and irradiate infra- and supraclavicular plus internal mammary nodes (standard). If extensive nodal involvement is present, the whole of the axillary region can be irradiated (option). Radiotherapy should only be omitted in the setting of a randomised clinical trial (option).

#### Palpable tumour, primary breast-conserving surgery not possible

**Figure 11:**
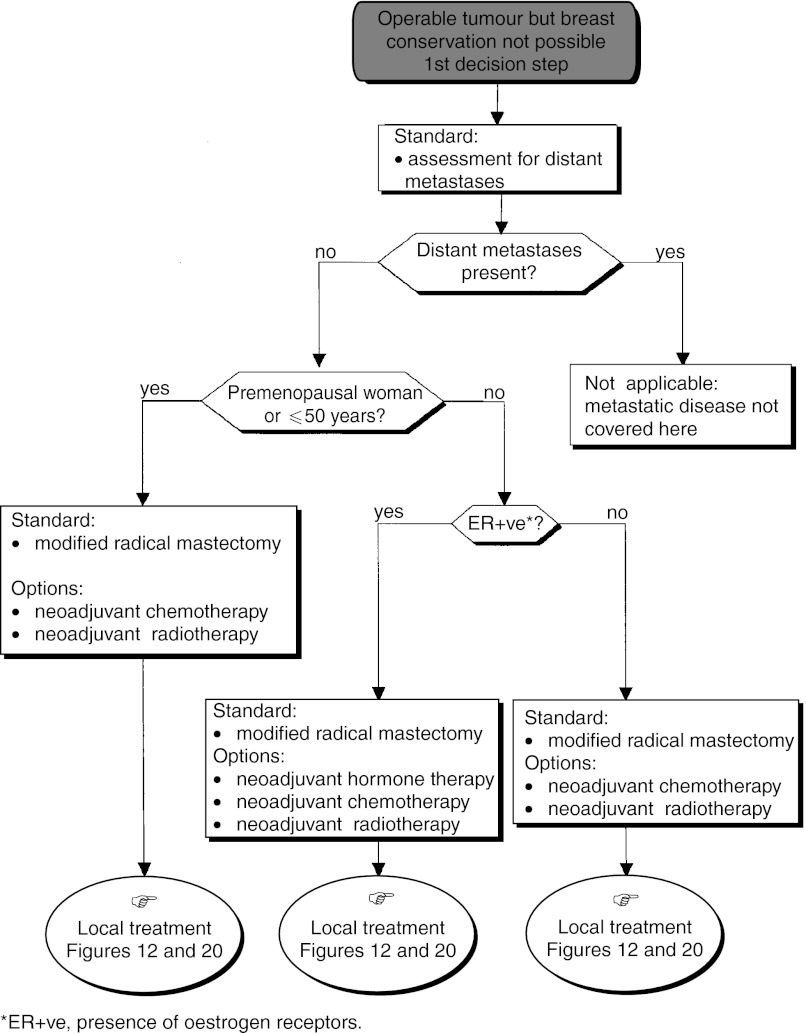
Palpable tumour, breast-conserving surgery not possible.

**Figure 12:**
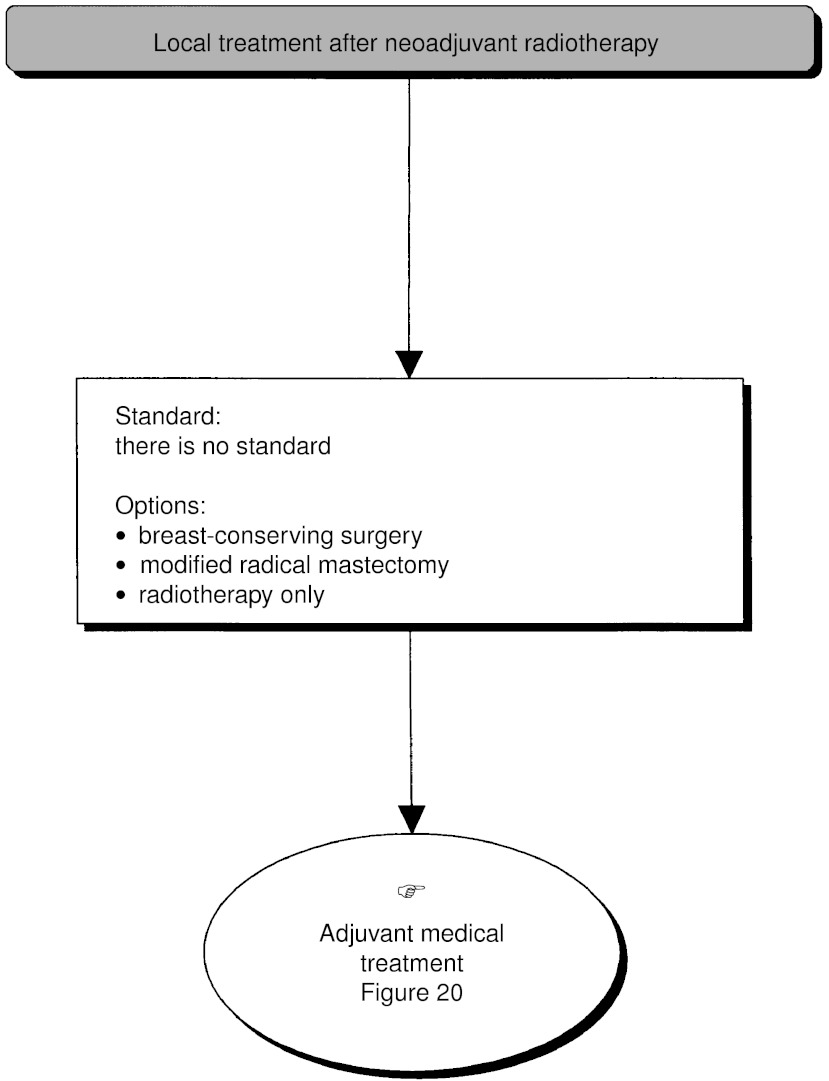
Palpable tumour, breast-conserving surgery not possible–local treatment after neoadjuvant radiotherapy.

**Figure 13:**
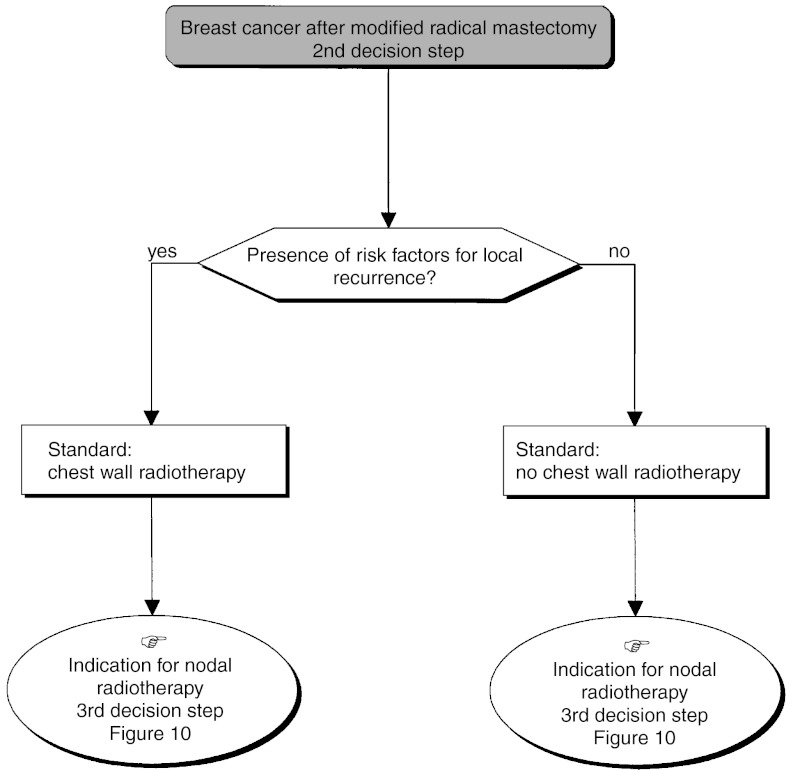
Palpable tumour, breast-conserving surgery not possible: modified radical mastectomy.

- *First decision step*: A metastatic screen should be undertaken (standard) (Figure 11

). A modified radical mastectomy should be performed in the absence of metastases (standard). Nonsurgical treatment (medical or radiotherapy) can be considered (option). Nonsurgical treatment is not indicated for multifoci lesions where the local treatment should be mastectomy (recommendation, expert agreement). If primary radiotherapy or medical treatment is performed, locoregional control must be obtained (recommendation, level of evidence: A) (Figure 12

). A preliminary biopsy can be performed to assess the prognostic factors that are necessary to guide locoregional and adjuvant treatment (recommendation, expert agreement). When immediate reconstruction is offered, it should not jeopardise the administration of locoregional and/or systemic treatment (recommendation).
- *Second decision step (*Figure 13

): This step occurs after mastectomy and axillary dissection and is dependent on the presence or absence of risk factors for local recurrence.
  ○ *Absence of risk factors for local recurrence*: Chest wall radiotherapy is not indicated (standard).
  ○ *Presence of risk factors for local recurrence*: Chest wall radiotherapy is indicated (standard).
- *Third decision step* (Figure 10): This step is dependent on the extent of axillary node involvement (see section on ‘Management of a single palpable localised tumour treatable by breast-conserving surgery, third decision step’).

### Adjuvant therapy (Figure 14)

**Figure 14:**
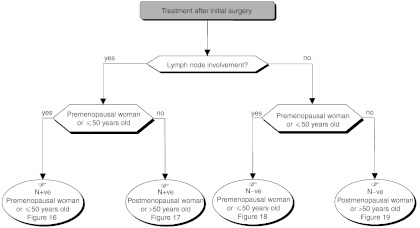
Operable tumour–adjuvant treatment.

The aim of adjuvant therapy is to reduce the risk of metastatic recurrence and thus improve survival. Risk factors for metastatic recurrence should be assessed during the initial examination and from the histopathological examination of surgical specimens. Adjuvant therapy should not replace optimal locoregional treatment. The patient's menopausal status and the tumour hormone receptor status should be used in the selection of one of the reference treatments: hormone therapy and/or chemotherapy.

#### Adjuvant therapy after surgery and initial axillary dissection with the possibility of breast conservation

The standards, options and recommendations are summarised in Table 3

**Table 3:** Management of patients with breast cancer with lymph node involvement (N+ve)

	**Premenopaual woman or ⩽50 years old (Figure 16)**		**Post menopausal woman or >50 years old (Figure 17)**	
	**ER+ve**	**ER−ve or unknown**	**ER+ve or unknown**	**ER−ve**
Standards	Chemotherapy and tamoxifen	Chemotherapy, no hormone therapy	Tamoxifen	No standard
				
Options	Chemotherapy and ovarian suppression ±tamoxifen		Tamoxifen and chemotherapy	Chemotherapy
	Ovarian suppression ±tamoxifen (no chemotherapy)			No adjuvant treatment
				
Recommendations	Tamoxifen as adjuvant treatment in premenopausal women can only be used in association with chemotherapy	Independent of the hormone receptor status of the tumour. High dose chemotherapy is not recommended except in the setting of a randomised clinical trial (expert agreement)		No chemotherapy if age and performance status suggest poor short-and/or long-term tolerance (level of evidence: B)
	The efficacy of the combination of ovarian suppression and antioestrogens is unknown (level of evidence: D) This combination should be evaluated in randomised clinical trials (expert agreement)			

ER+ve=presence of oestrogen receptors; ER−ve=absence of oestrogen receptors.

and 4

**Table 4:** Management of patients with breast cancer without lymph node involvement (N−ve)

	**Premenopausl woman or ⩽50 years old (Figure 18)**			**Postmenopausal woman or >50 years old (Figure 19)**		
	**Risk of metastatic recurrence^a^**		**No risk of metastatic recurrence**	**Risk of metastatic recurrence^a^**		**No risk of metastatic recurrence**
	**ER+ve**	**ER−ve or unknown**		**ER+ve or unknown**	**ER−ve**	
Standards	Chemotherapy and tamoxifen	Chemotherapy no hormonotherapy (level of evidence: B)	No adjuvant treatment	Tamoxifen	No standard	No adjuvant treatment
						
Options	Chemotherapy and ovarian suppression, and tamoxifen		Tamoxifen if ER+ve	Tamoxifen and chemotherapy	Chemotherapy	Tamoxifen if ER+ve
	Ovarian suppression ±tamoxifen (no chemotherapy)				No adjuvant medical treatment	
						
Recommendations				Combination of tamoxifen and chemotherapy in the setting of randomised clinical trials (level of evidence: B)		

a Presence of one or more risk factors for metastatic recurrence.

ER+ve=presence of oestrogen receptors; ER−ve=absence of oestrogen receptors.

and Figure 15

**Figure 15:**
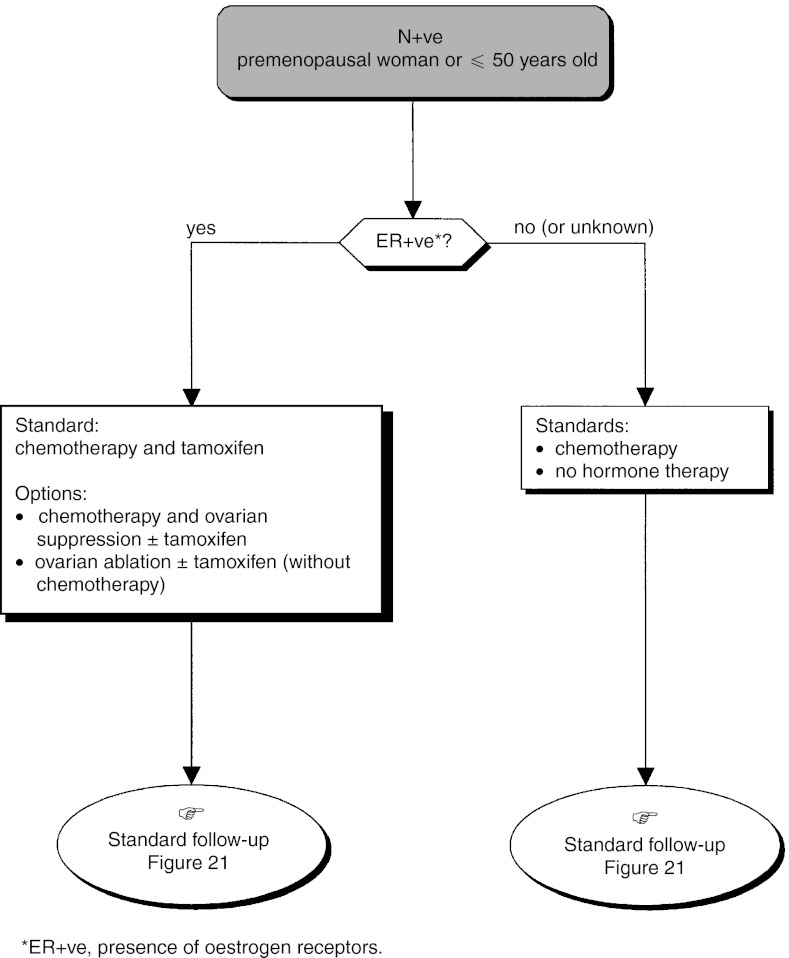
Operable tumour–adjuvant medical treatment (N+ve, premenopausal woman or ⩽50 years old).

, 16

**Figure 16:**
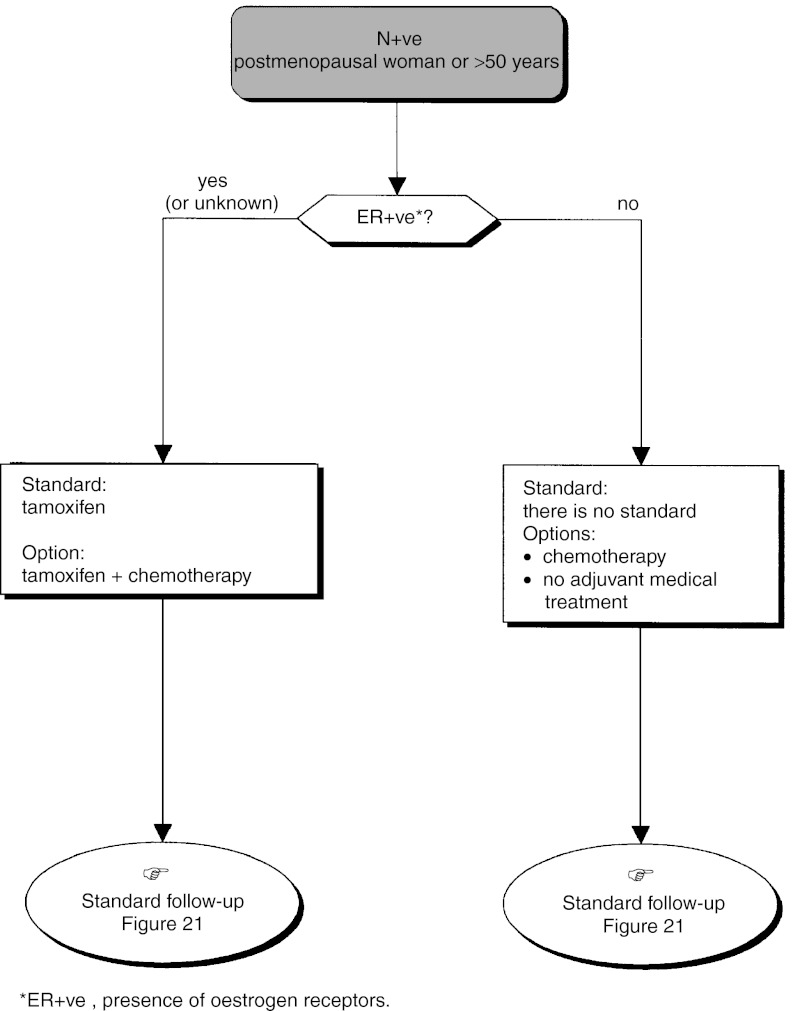
Operable tumour–adjuvant treatment (N+ve, postmenopausal woman or >50 years old).

, Figure 17

**Figure 17:**
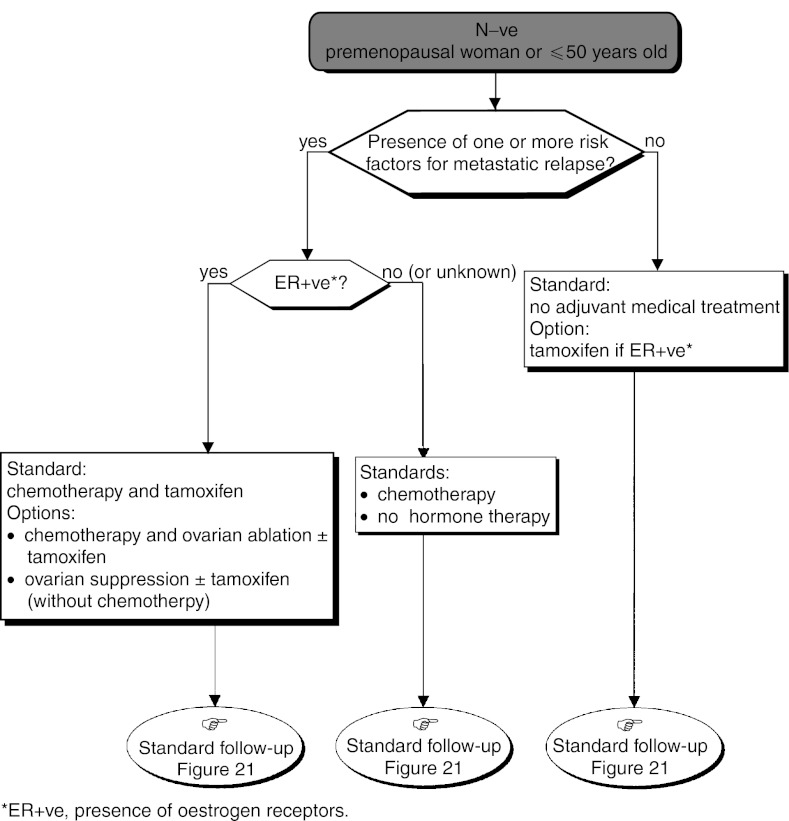
Operable tumour–adjuvant medical treatment (N−ve, premenopausal woman or ⩽50 years old).

and Figure 18

**Figure 18:**
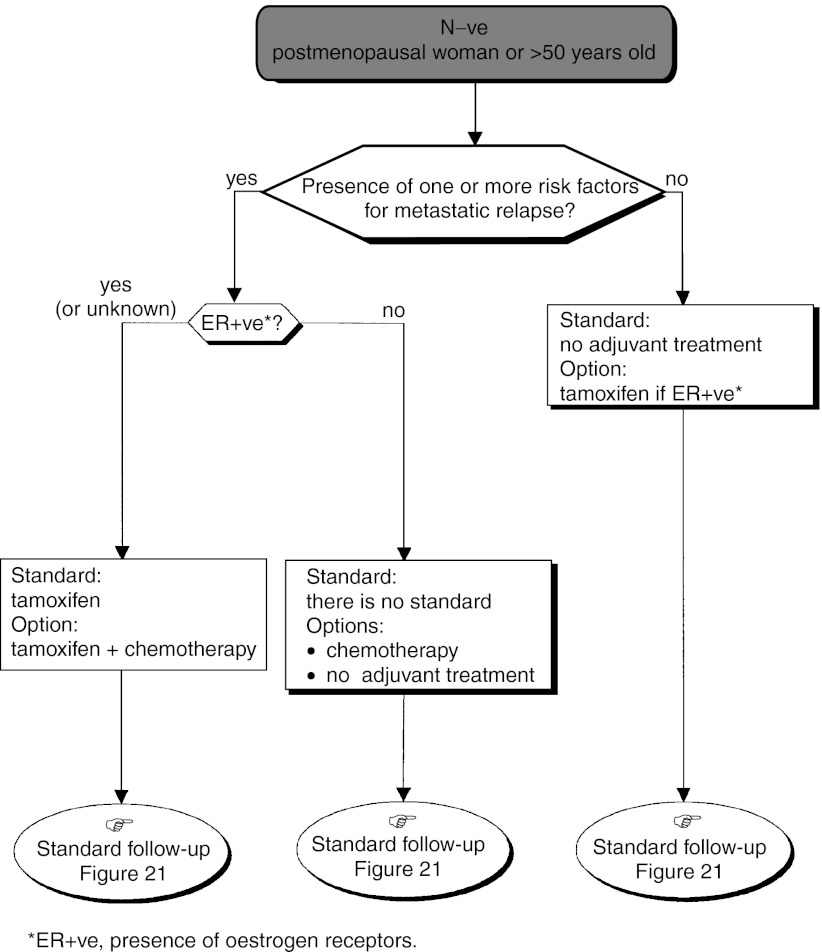
Operable tumour–adjuvant medical treatment (N−ve, postmenopausal woman or >50 years old).

.

#### Adjuvant therapy after first-line chemotherapy or hormone therapy

**Figure 19:**
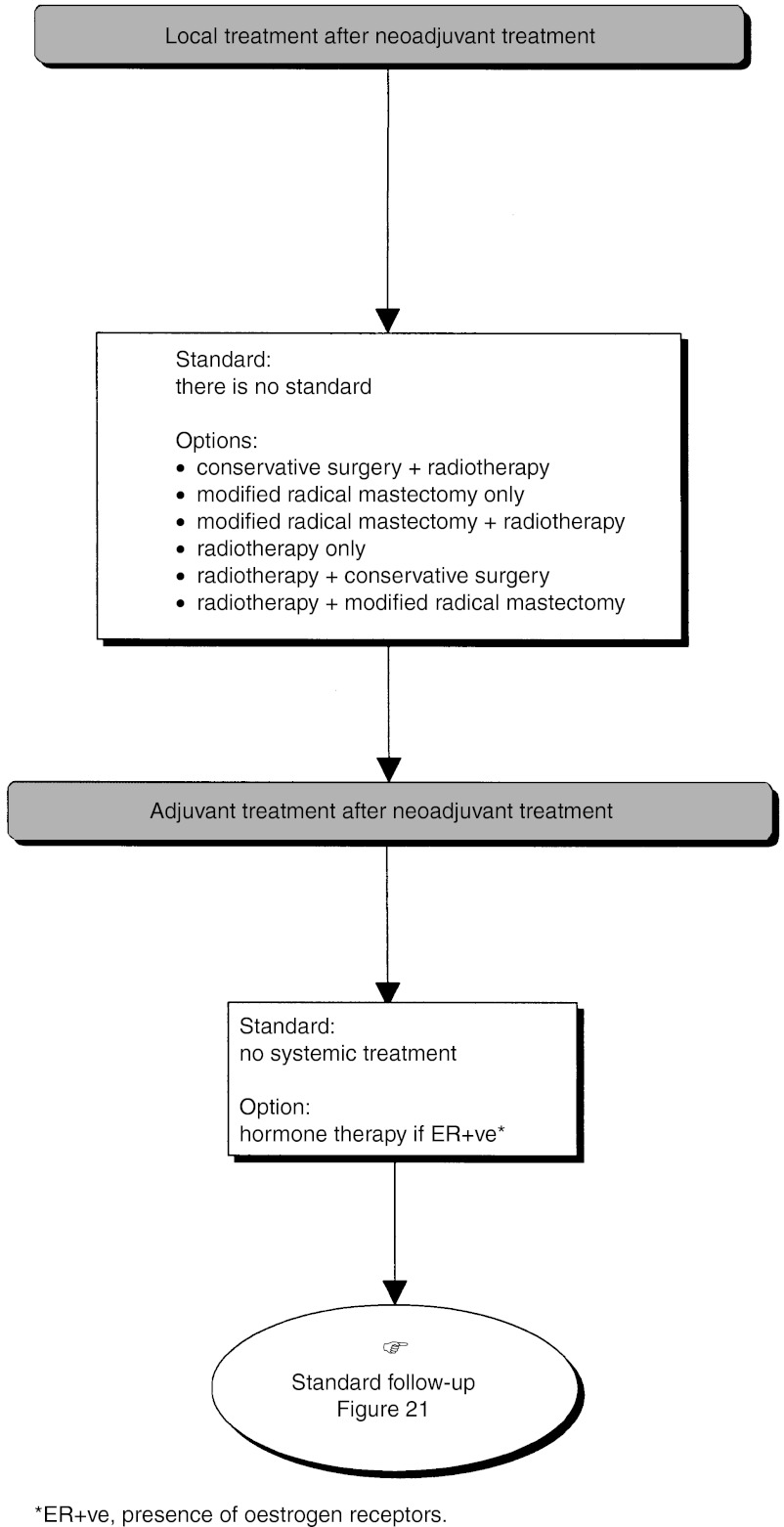
Palpable tumour, breast-conserving surgery not possible–local treatment after neoadjuvant treatment.

There is no standard treatment. Hormone therapy with tamoxifen can be given if the tumour is positive (or unknown) for oestrogen receptors (option) (Figure 19

).

#### Adjuvant therapy after first-line radiotherapy

**Figure 20:**
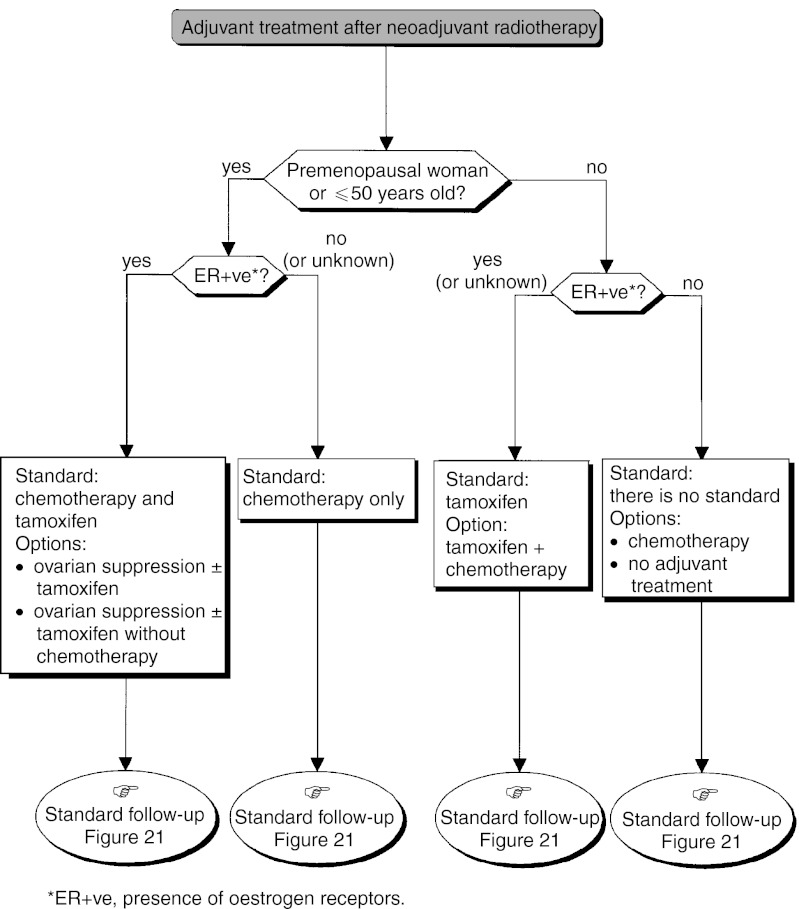
Palpable tumour, breast-conserving surgery not possible–local treatment after neoadjuvant radiotherapy.

The size of the initial lesion may justify the use of adjuvant medical therapy (recommendation) (Figure 20

).

## FOLLOW-UP

**Figure 21:**
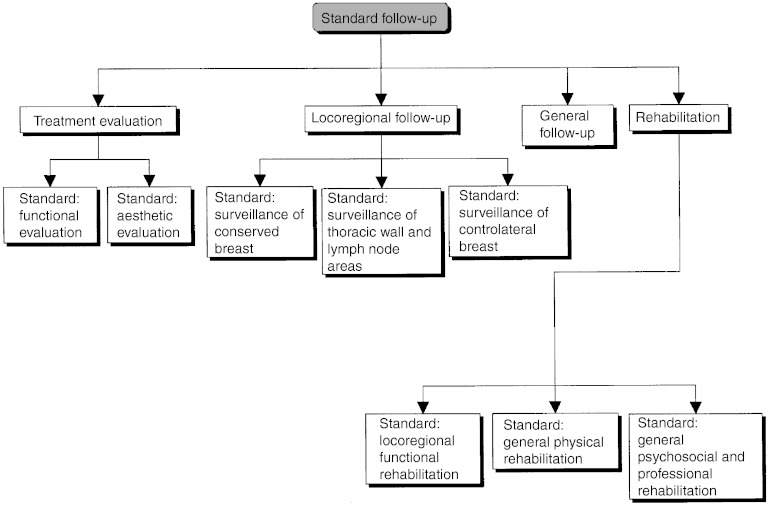
Standard follow-up.

Follow-up should focus on the evaluation of the treatment results, screening for relapse, treatment of side effects and psychosocial and professional rehabilitation (Figure 21

). This requires a multidisciplinary approach.

### Follow-up of the conserved breast

Clinical examination should be performed 4 months after treatment to assess treatment toxicity (standard). Clinical follow-up should then be repeated every 6 months for 5 years and then yearly for 10 years (standard). Routine follow-up should be continued after 10 years, but the timing can be adjusted depending on the risk of local recurrence (option). An annual mammogram should be performed starting 6 months after treatment (standard).

### Follow-up of the chest and lymph node areas

Routine clinical examination forms the basis of follow-up for the thoracic wall and the lymph node areas after treatment for breast cancer.

### Follow-up of the contralateral breast

Clinical examination and a mammogram should be performed at the same frequency as above for the follow-up of the conserved breast (standard, expert agreement).

### General follow-up after treatment of patients in complete remission

History taking and clinical examination form the basis of follow-up (standard). In the absence of symptoms or signs, a routine screen for metastases is not indicated (standard, level of evidence: A). If a metastasis is found, the patient should undergo a full work-up (recommendation, expert agreement). The frequency of general clinical follow-up is the same as that for locoregional follow-up (recommendation, expert agreement).

### Rehabilitation after treatment for breast cancer

Rehabilitation should start before treatment with clear specific information about possible post-treatment complications and how they can be prevented and/or managed (standard). Functional problems of arm or shoulder movement require early physiotherapy (standard). Lymphoedema can be treated with physical methods and systemic therapy (recommendation). Coumarin is no longer indicated because of its toxicity and lack of efficacy (standard).

To avoid weight gain, dietary advice should be provided routinely (recommendation). Sexual problems should be evaluated and treated (recommendation). The need for contraception and family planning advice should be discussed individually taking into consideration each patient's preference (recommendation).

Hormone replacement treatment for postmenopausal symptoms should not be prescribed after treatment for breast cancer, except in specific cases. The prescription of hormone replacement treatment after breast cancer is being evaluated prospectively. Nonhormonal treatments exist for the various symptoms (recommendation).

Psychological support may well be required at some stage during the management process (option). Social support, to strengthen the psychological support, should be provided routinely to help patients and their families (option). Patient rehabilitation groups can contribute to the psychosocial support of patients (option).

### Management of patients with recurrent disease

#### Management of local recurrence after breast-conserving treatment for breast cancer

Local recurrence should be treated with surgery. Radiotherapy should not be considered except in specific cases (standard). The standard treatment is a simple total mastectomy (standard). Immediate reconstruction can be considered (option). If oestrogen receptors are present, additional hormone therapy is recommended (recommendation).

Breast-conserving surgery can only be considered if the patient refuses mastectomy or if mastectomy is technically impossible. In this case, the patient should be informed of the high risk of recurrent disease (recommendation). As the efficacy of additional chemotherapy is unknown, this should only be considered in the setting of a randomised clinical trial (recommendation, expert agreement).

#### Management of uncontrolled, isolated local recurrence (inflammatory or locally advanced tumour)

There is no standard (standard). Chemotherapy can be considered, followed if possible, by local treatment that may or may not be curative (options). Local treatment (surgery and/or radiotherapy) for symptom control can also be considered (option).

Chemotherapy is appropriate for an inflammatory recurrence. Radiotherapy can be considered if there are contraindications for chemotherapy (option). When possible, mastectomy should be performed for a locally advanced recurrence (option). If this is not possible, chemotherapy or radiotherapy should be given with the aim of making the lesion operable (option). Irrespective of the treatments administered, the indications for additional medical treatment will be the same as those for potentially curable isolated recurrences (recommendation, expert agreement).

## Appendix

### Reviewers

J Alexandre (Hôpital Paul Brousse, Villejuif), P Allain (Polyclinique du Parc, Caen), C Allavena (Centre Sainte Catherine de Sienne, Nantes), M Antoine (Hôpital Tenon, Paris), P Banzet (Hôpital Saint-Louis, Paris), JP Basuyau (Centre Henri-Becquerel, Rouen), J Becue (CHRU - Hôpital Rangueil, Toulouse), JP Bergerat (CHRU, Strasbourg), P Bey (Centre Alexis Vautrin, Nancy), YJ Bignon (Centre Jean-Perrin, Clermont-Ferrand), P Bougnoux (CHU Hôpital Bretonneau, Tours), A Brémond (Centre Régional Léon Bérard, Lyon), J Chauvergne (Institut Bergonié, Bordeaux), J Cuminet (Hôpital Saint Louis, Paris), A Daver (Centre Paul Papin, Angers), B De Korvain (CHRU Hôpital Sud, Rennes), L Demange (Polyclinique de Courlancy, Reims), JL Demeaux (Bordeaux), M Durand (Institut Bergonié, Bordeaux), E Fondrinier (Centre Paul Papin, Angers), J Gligorov (Hôpital Tenon, Paris), MJ Goudier (Hôpital Bodelio, Lorient), O Jourdain (Clinique Jean Villars, Bruges), P Kerbrat (Centre Eugène Marquis, Rennes), Y Kessler (Polyclinique de Gentilly, Nancy), J Lalaude (CH de Falaise, Falaise), O Lefloch (Hôpital Bretonneau, Tours), J Lévêque (CHRU Hôpital Sud, Rennes), A Lortholary (Centre Paul-Papin, Angers), A Meunier (Médipole Gentilly Saint Jacques, Maxeville), MP Meurisse (CHG Hôpital Robert Boulin, Libourne), G Milano (Centre Antoine-Lacassagne, Nice), T Petit (Centre Paul Strauss, Strasbourg), T Philip (Centre Léon Bérard, Lyon), G Prévot (CH Hôpital du Hasenrain, Mulhouse), D Querleu (Centre Oscar Lambert, Lille), JF Ribot (Clinique du Pont de Chaume, Montauban), P Romestaing (CH Lyon Sud, Pierre Bénite), J Rouëssé (Centre René-Huguenin, Saint-Cloud), S Scholl (Institut Curie, Paris), J Stines (Centre Alexis Vautrin, Nancy), JF Sztermer (CH Jean-Monnet, Epinal), H Tristant (Institut de radiologie, Paris), P Troufleau (Centre Alexis Vautrin, Nancy), D Vaillant (Centre Georges-François Leclerc, Dijon), JL Verhaeghe (Centre Alexis Vautrin, Nancy), R Villet (Hôpital Diaconesses, Neuilly sur Seine), JJ Voigt (Institut Claudius Régaud, Toulouse), D Zylberait (CHG de Compiègne, Compiègne).
